# Reversing inflammatory diseases via trained immunity: mechanisms, challenges, and prospects

**DOI:** 10.3389/fimmu.2025.1666233

**Published:** 2025-10-15

**Authors:** Wanli Xu, Zhilin Guo, Tingyun Xu, Junjie Chen, Leyi Chen, Wenan Xu

**Affiliations:** ^1^ Shenzhen Clinical College of Stomatology, School of Stomatology, Southern Medical University, Shenzhen, Guangdong, China; ^2^ Shenzhen Stomatology Hospital (Pingshan) of Southern Medical University, Shenzhen, Guangdong, China; ^3^ School of Stomatology, Southern Medical University, Guangzhou, Guangdong, China

**Keywords:** trained immunity, inflammatory diseases, immune system, innate immune memory, epigenetic reprogramming, inflammation

## Abstract

Chronic inflammatory diseases are widespread and often accompanied by comorbidities, making treatment challenging. Current immunosuppressive and anti-inflammatory therapies have limited efficacy and significant side effects, and are insufficient to address the complexity of coexisting conditions. This review explores recent advances in innate immune memory, also known as trained immunity, and its potential role in inflammatory diseases. We hypothesize that targeting the regulatory mechanisms of trained immunity may lead to novel therapeutic strategies that more effectively control inflammation and improve disease outcomes. Finally, we highlight that the interplay between trained immunity and inflammatory diseases remains incompletely understood, and further research is needed to elucidate its mechanisms and clinical translational potential.

## 1 Introduction

With the shifting landscape of modern disease burden, chronic inflammation-related disorders have emerged as a major health concern. Inflammatory diseases are central to a wide spectrum of conditions, including infections, autoimmune disorders, and metabolic syndromes, and are frequently accompanied by chronic comorbidities, forming complex pathological networks. Current therapeutic strategies, such as immunosuppressants, nonsteroidal anti-inflammatory drugs, and biologics, offer partial symptom relief but are often limited by suboptimal efficacy, considerable side effects, and risks of immune suppression-related complications. Particularly under the coexistence of Multiple chronic inflammatory diseases, treating a single disease entity often fails to achieve durable outcomes, highlighting the urgent need for novel therapeutic perspectives.

Recent advances in immunology have challenged the conventional view that immunological memory is exclusive to the adaptive immune system. It is now recognized that the innate immune system also possesses a form of memory-like response, termed trained immunity (1). This phenomenon was first identified in plants and invertebrates, which lack adaptive immunity, and is characterized by enhanced protection upon secondary challenge following a primary encounter. Trained immunity has since been observed in vertebrates as well, suggesting that it represents an evolutionarily conserved defense strategy. Mechanistically, trained immunity is mediated through epigenetic reprogramming, enabling innate immune cells to mount faster and more robust responses upon restimulation.

However, trained immunity presents a double-edged sword. While it confers beneficial effects in host defense against pathogens, its excessive activation may exacerbate pathological inflammation, thereby contributing to the progression of inflammatory diseases. Emerging evidence indicates that features of aberrant trained immunity are present in patients with inflammatory disorders and chronic comorbidities, providing a theoretical framework for reinterpreting disease mechanisms from an innate immune memory perspective. This review aims to provide a comprehensive overview of the concept and evolutionary origins of trained immunity, examine its roles in inflammatory diseases, and propose novel therapeutic approaches targeting trained immunity. By precisely modulating the activation state of trained immunity, it may be possible to suppress pathological inflammation while preserving essential immune defenses, offering promising new avenues for the treatment of inflammation-related diseases and chronic complications.

## 2 Another memory of immune system: trained immunity

The human immune system is categorized into innate and adaptive immunity. During inflammation, pathogen invasion is initially countered by physical barriers such as the skin and mucosal surfaces. Once pathogens breach these barriers, innate immune cells and molecules are rapidly activated, triggering the innate immune response. This leads to the immediate release of cytokines and chemokines, which recruit and activate immune cells such as neutrophils and macrophages to eliminate pathogens (2, 3). Subsequently, dendritic cells and macrophages present pathogen-derived antigens to T cells, initiating adaptive immune responses. Activated B cells produce specific antibodies, and immunological memory is established to enhance protection against future infections with the same pathogen (4). Historically, research has predominantly focused on adaptive immune memory. However, emerging evidence indicates that, beyond T and B lymphocytes, innate immune cells and even non-immune cells exhibit a memory-like response. Upon encountering microbial components or their products, these cells mount a faster and more robust reaction upon secondary exposure. This phenomenon suggests the existence of memory within the innate immune system. In 2011, Mihai Netea defined this biological process as “trained immunity” (5), while in 2017, Elaine Fuchs referred to it as “inflammatory memory” (6). Unlike the lifelong persistence of adaptive immune memory, trained immunity is transient, typically lasting from at least 3 months to up to 1 year (7). Notably, heterologous protection induced by live vaccines may persist for as long as 5 years (8). Recent studies also reveal that this immunological phenotype can be transmitted across generations (9). In contrast to the recombination-driven mechanisms of adaptive memory, trained immunity is mediated through reversible epigenetic modifications, including chromatin remodeling, long non-coding RNA (lncRNA) transcription, DNA methylation, and metabolic reprogramming. These alterations partly persist after the cessation of stimulation, thereby enabling a form of functional memory. Furthermore, while adaptive memory enhances responses to specific antigens, trained immunity provides broad-spectrum protection against diverse pathogens in a non-specific manner (10). In summary, both innate and adaptive immunological memory collaboratively maintain host defense and homeostasis.

## 3 The origin of trained immunity

This phenomenon of non-specific immune memory was first observed in plants and invertebrates, both of which, as evolutionarily ancient organisms, lack adaptive immune systems and rely solely on innate immune mechanisms to combat infections (11), does this imply that they possess immune memory? Upon exposure to attenuated microbes, plants can develop long-lasting, broad-spectrum resistance to various pathogens, including viruses, bacteria, fungi, and oomycetes (12). Systemic acquired resistance (SAR) is induced following pathogenic infection and spreads from the infection site to the entire plant. This process is mediated by signaling molecules (such as salicylic acid and jasmonic acid), as well as regulatory factors (such as NPR1 and SNI1), thereby establishing a systemic defense response (12). SAR represents a “memory-like” defensive strategy in plants, offering long-term protection across the whole organism, analogous to immunological memory in animals (13). These findings suggest that plants can also acquire memory capacity through innate immune pathways. Similarly, Drosophila exhibits protection against reinfection with the same pathogen after prior exposure to *Streptococcus pneumoniae* or *Beauveria bassiana* (14). Mosquitoes infected with plasmodium falciparum show partial protection upon re-infection (15). Moreover, water fleas (Daphnia) can transfer pathogen-specific immunity against *pasteuria ramosa* to their offspring via maternal transmission (16). Other invertebrates such as sponges, corals, small crustaceans, and shrimp also display features of immune memory, evidenced by stronger responses upon secondary challenge with the same donor (17, 18). These examples demonstrate that, despite lacking adaptive immune cells like T and B lymphocytes, invertebrates can still mount memory-like responses through their innate immune systems, offering protection upon re-exposure to identical or different pathogens. Such findings challenge the traditional view that innate immune responses are entirely non-adaptive and devoid of memory. The first vertebrate evidence of innate immune memory dates back to 1986, when researchers injected an avirulent, non-germinating strain of *Candida albicans* (PCA-2) into *Rag1^−/−^ mice* lacking mature T and B cells, these mice exhibited protection against subsequent lethal systemic C. albicans infection and also showed cross-protection against *Staphylococcus aureus*, this protection was independent of T-cell-mediated mechanisms and instead relied on innate immune components, particularly macrophages and cytokine production (19). Further, the non-specific protective effects of Bacillus Calmette–Guérin (BCG) vaccination have significantly advanced the study of trained immunity. BCG, a live attenuated vaccine derived from *Mycobacterium bovis*, is widely used to prevent tuberculosis (20). In murine models, BCG vaccination has been shown to confer protection against secondary infection with *Candida albicans* (21). Beyond experimental models, epidemiological studies have reported that BCG vaccination in children not only protects against tuberculosis but also reduces overall morbidity and mortality from unrelated infections (22). In regions with high tuberculosis prevalence, widespread BCG vaccination not only helps curb tuberculosis transmission but may also offer collateral protection against other infectious diseases.

**Figure 1:**
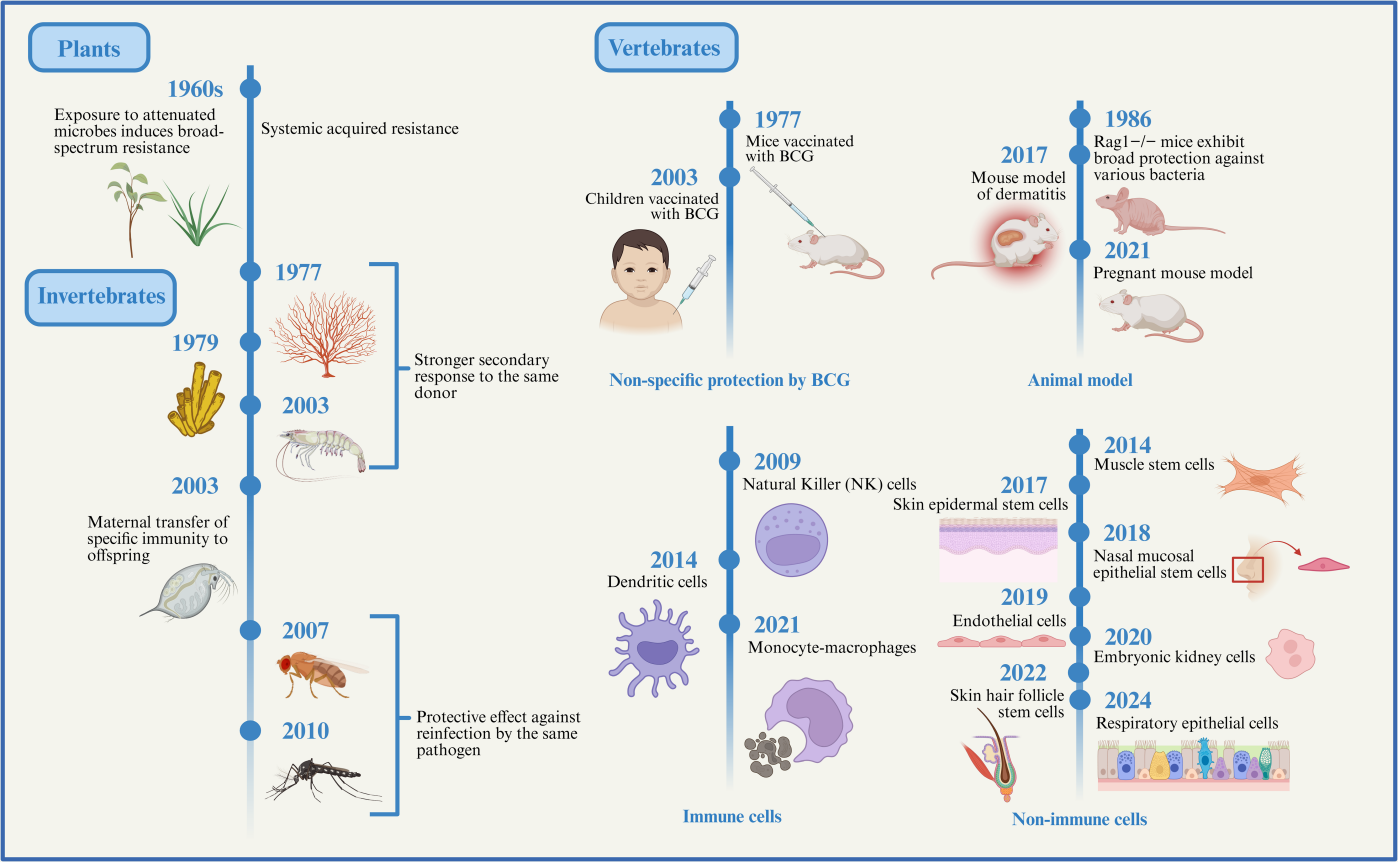
The evolution of trained immunity. Here, we outline the evolutionary background of innate immune memory. Although plants and invertebrates lack adaptive immune systems, their innate immune systems can still generate memory effects against prior infections, providing a certain degree of protection upon subsequent encounters with the same or different pathogens. Subsequently, studies on vertebrate animal models, immune cells, and non-immune cells have gradually revealed the existence and mechanisms of trained immunity, indicating that this form of immune memory has progressively developed and matured throughout evolution. Created with BioRender.com.

With the in-depth exploration of the non-specific protective effects of the BCG vaccine, it has become increasingly evident that the innate immune system is not devoid of memory capabilities, as traditionally believed. Instead, it possesses a certain degree of immune memory. This discovery has sparked widespread interest in trained immunity research in vertebrates. Researchers have begun investigating the forms, mechanisms, and potential applications of trained immunity in vertebrates from various perspectives and using multiple approaches (**Figure 1**). For example, the skin of mice previously exposed to inflammatory stimuli heals significantly faster upon subsequent injury compared to that of naïve mice (6). Offspring of pregnant mice that experienced infection exhibit stronger resistance to intestinal infections but are also more susceptible to intestinal inflammation (9). Lipopolysaccharide (LPS) induced trained immunity in lung-resident cells has been shown to confer robust protection against Streptococcus pneumoniae infection in mice (23). Subsequent studies have further explored the extensive cellular-level functions of trained immunity. Monocytes, macrophages, dendritic cells (DCs), and natural killer (NK) cells, all key components of the innate immune system, have recently been found to not only serve basic immune defense roles but also to exhibit memory-like characteristics. For instance, trained monocytes and macrophages produce higher levels of reactive oxygen species (ROS) and pro-inflammatory cytokines upon re-stimulation, enhancing their ability to initiate and sustain inflammatory responses and eliminate pathogens more effectively (24). NK cells expressing Ly49H expand rapidly following murine cytomegalovirus (MCMV) infection and demonstrate enhanced production of interferon-γ (IFN-γ) and stronger degranulation upon re-encounter with MCMV (25). Trained DCs have also been shown to process and present antigens more efficiently to T lymphocytes, thereby facilitating adaptive immune activation (26). Thus, innate and adaptive immunity act synergistically to protect the host. Interestingly, trained immunity is not limited to immune cells. Recent research shows that non-immune cells can also exhibit trained immunity. Inflammation induced by skin injury prompts epidermal stem cells (EpSCs) to retain long-term memory that accelerates wound healing upon subsequent injury (6). Hair follicle stem cells are capable of differentiating into epidermal cells while still maintaining their ability to produce hair after epidermal regeneration (27). The trained immunity-like behavior of EpSCs is particularly significant, as tissue stem cells are central to maintaining homeostasis and regeneration through sensing environmental cues and adjusting their behavior. Moreover, *S. pneumoniae* infection induces memory in respiratory epithelial cells, which enhances bacterial adhesion and facilitates infection upon reinfection (28). Additional studies have reported the presence of trained immunity in muscle stem cells, intestinal epithelial cells, and nasal mucosal epithelial stem cells (9, 29, 30). These findings collectively support the notion that the human immune system harbors a more primitive, innate form of immune memory.

## 4 The dual role of trained immunity

Trained immunity may represent an evolutionarily primitive form of immune memory. It is a remarkable capacity of the body to recall prior adverse encounters and respond more swiftly upon re-exposure, thereby enhancing host defense and survival. For instance, pancreatic epithelial cells that recover from acute inflammation can rapidly reinitiate acinar-to-ductal metaplasia (ADM) during subsequent inflammatory events, effectively limiting tissue damage by promptly reducing zymogen production (31). Similarly, maternal exposure to inflammatory insults during pregnancy leads to elevated IL-6 levels in the systemic circulation, which induces IL-6R expression in fetal intestinal epithelial stem cells, this inflammatory imprint persists into adulthood, conferring increased resistance to intestinal infections in the offspring (9). In addition to epithelial barriers in the digestive tract, other barrier tissues benefit from trained immunity through enhanced tissue repair and antimicrobial capacity. Acute exposure to LPS induces long-lasting changes in airway macrophages, resulting in an innate immune memory phenotype that protects against bacterial pneumonia (23). Likewise, brief cutaneous exposure to imiquimod, an acute inflammatory trigger that activates IL-17-type psoriasis-like responses, enhances wound healing capacity thereafter (6). Protective effects of trained immunity have also been reported in various *in vivo* models. Pre-injection of β-glucan in mice significantly improves survival against *S.aureus* and reduces renal necrosis associated with systemic infection (32). In leukemic mice, β-glucan not only prolongs survival but also extends lifespan during experimental *S. aureus* sepsis (33). Intraperitoneal administration of cytosine–guanine (CpG) oligodeoxynucleotides confers protection against Escherichia coli-induced meningitis in neutropenic mice (34). Interestingly, compared with sedentary controls, bone marrow-derived macrophages (BMDMs) from exercise-trained mice show reduced LPS-induced NF-κB activation and proinflammatory gene expression, along with increased expression of M2-related genes, these changes are associated with improved mitochondrial quality, increased reliance on oxidative phosphorylation, and reduced ROS production (35, 36). These findings suggest that moderate, regular exercise may activate trained immunity, strengthening host defense while reducing chronic inflammation and disease incidence.

**Figure 2:**
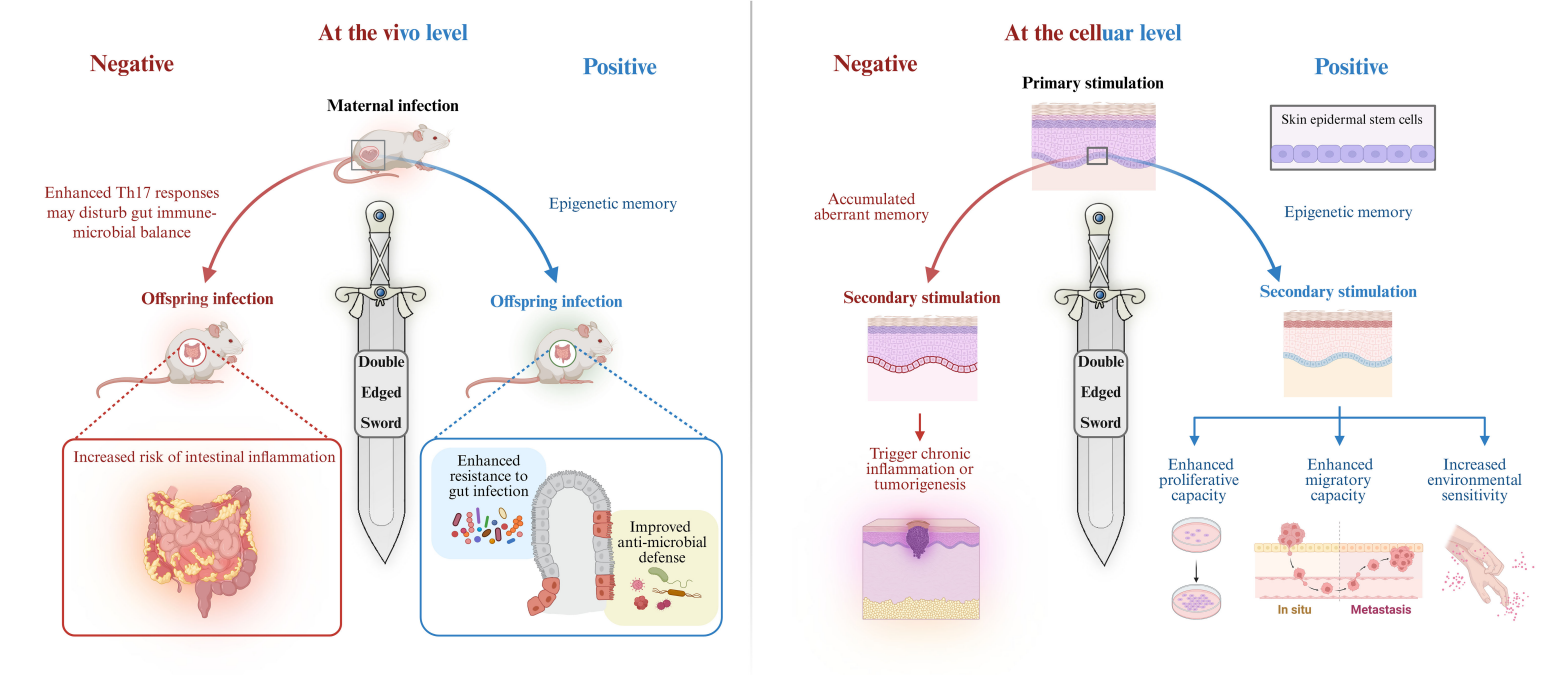
Trained immunity is a double-edged sword. Trained immunity plays a dual role in inflammation regulation and immune defense. At the organismal level, maternal inflammation during pregnancy elevates IL-6 levels, activating IL-6R expression in fetal intestinal epithelial stem cells and establishing lasting inflammatory memory. This memory enhances offspring resistance to intestinal infections. On the other hand, enhanced Th17 responses may disrupt the balance between the gut microbiota and the immune system, causing immune overactivation and chronic inflammation, thereby increasing the risk of intestinal inflammatory diseases in the offspring (9). At the cellular level, transient inflammatory stimulation by imiquimod endows EpSCs with memory capacity, promoting proliferation and migration, enhancing wound healing, and strengthening resistance to future pathogens, thereby providing protection. However, prolonged excessive stimulation of stem cells may lead to “overtrained,” accumulate abnormal memories, excessive proliferation and migration, and ultimately triggering chronic inflammation or tumorigenesis (6). Created with BioRender.com.

In contrast, chronic excessive inflammation or tissue injury represents another potential consequence of trained immunity, contributing to the pathogenesis of diseases such as atherosclerosis, rheumatoid arthritis, psoriasis, and inflammatory bowel disease. In these contexts, the maladaptive effects of trained immunity may exacerbate disease pathology. During tissue repair, skin stem cells retain epigenetic memory associated with migration and inflammation, endowing them with enhanced proliferative capacity, increased migratory ability, and heightened environmental sensitivity, these traits facilitate more efficient regeneration following recurrent injuries (37). However, it is noteworthy that such regenerative features significantly overlap with malignant characteristics of tumor cells, including uncontrolled proliferation and invasive migration (38), thereby potentially promoting tumor initiation and metastasis. Moreover, the persistent accumulation of inflammatory memory may lead to stem cell dysfunction, resulting in exaggerated immune responses and increased susceptibility to autoimmune diseases (e.g., psoriasis) and chronic inflammatory conditions. Over time, the abnormal buildup of epigenetic memory may become a key contributor to chronic inflammation and tumorigenesis. Similarly, although maternal inflammatory training of intestinal stem cells offers infection resistance to offspring, the concurrently heightened Th17 response may disrupt host–microbiota homeostasis, triggering immune overactivation and chronic inflammation, and thereby elevating the risk of intestinal inflammatory disorders in the progeny (9) (**Figure 2**). These findings suggest that while acute and transient injury can potentiate repair mechanisms, repeated insults may drive chronic inflammation and impair healing. Thus, trained immunity is a double-edged sword, its net effect depends on whether it enhances or aggravates the tissue response.

## 5 Trained immunity of inflammatory diseases

### 5.1 Autoimmune diseases

#### 5.1.1 Sarcoidosis

Sarcoidosis is a non-caseating epithelioid granulomatous inflammatory disease, the formation of these granulomas disrupts tissue homeostasis, primarily affecting the lung parenchyma and leading to clinical symptoms such as cough and dyspnea, Multiple organ involvement (39). Despite the identification of various environmental, occupational, infectious, and genetic risk factors, the precise etiology of sarcoidosis remains unclear. It is thought to involve aberrant immune responses but is not classified as a typical autoimmune disorder. Glucocorticoids remain the first-line therapy, but their use carries a dose-dependent risk of severe adverse events (40). Anti-TNF-α biologics are effective in steroid-refractory or organ-threatening cases. For severe and progressive disease, third-line treatment may include biologics or advanced immunomodulators (40). Sarcoid granulomas consist of both immune and structural cells. Their formation begins when macrophages and dendritic cells recognize antigens via pattern recognition receptors (PRRs), triggering the secretion of cytokines (e.g., TNF-α, IL-1β, IL-6, IL-12) and chemokines (e.g., CXCL9, CXCL10, CCL2), these mediators promote macrophage recruitment and aggregation to form the granuloma core (41). Th17.1 and Th1 cells contribute to granuloma development and maintenance by secreting IL-17 and IFN-γ, thereby regulating immune cell recruitment and granuloma architecture (42). The granuloma center typically comprises CD68^+^ macrophages and multinucleated giant cells with M1-like characteristics, while the periphery is enriched in Th1 and Th17.1 cells (43). Together, these components sustain granulomatous inflammation and perpetuate the disease process.

Currently, limited evidence supports a direct role for trained immunity in sarcoidosis, However, some studies suggest that monocytes in sarcoidosis patients exhibit pathological features overlapping with the detrimental effects observed in trained immunity. Pathogen exposure may trigger trained immunity in sarcoidosis, peripheral blood mononuclear cells (PBMCs) from patients produce higher TNF and IL-6 levels upon bacterial or fungal stimulation than those from healthy controls and show abnormal expression of pathogen-sensing PRRs (44). metabolic alterations in monocytes have been identified as another hallmark, PBMCs from sarcoidosis patients show dysregulation of metabolic and oxidative phosphorylation pathways, particularly upregulation of the mechanistic target of rapamycin (mTOR) signaling pathway (45). Hyperactivation of mTOR promotes macrophage proliferation, inhibits apoptosis, and drives metabolic reprogramming, thereby supporting macrophage survival and granuloma persistence (46, 47). mTORC1 maintains granuloma structure, while JAK/STAT enhances cytokine production and immune cell activation, they suppress macrophage autophagy and promote formation of multinucleated giant cells, a hallmark of sarcoid granulomas (48). Downstream of mTOR, hypoxia-inducible factor-1α (HIF-1α) is excessively activated in sarcoid macrophages, exacerbating inflammation and fibrosis (49). Dysregulated mTOR and HIF-1α signaling disrupt glycolysis and impair the tricarboxylic acid (TCA) cycle, a metabolic cascade implicated in protective β-glucan-induced trained immunity (50). Metabolomic analysis of sarcoidosis patient serum reveals perturbations in glycolysis and TCA cycle activity, contributing to macrophage hypertrophy and multinucleated giant cell formation—the key components of granulomas. Activated pentose phosphate pathway (PPP) and elevated metabolic states in monocytes further correlate with granulomatous inflammation (43).

The precise epigenetic mechanisms in sarcoidosis remain poorly defined. However, bronchoalveolar lavage cells from sarcoidosis patients show aberrant DNA methylation in genes linked to immune responses, such as *HLA-DPB2, CXCL7, and CCL16*, suggesting dysregulated gene expression due to DNA methylation and chromatin remodeling (51, 52). These metabolic and potential epigenetic alterations may amplify monocyte dysfunction in sarcoidosis.

Although current studies on trained immunity in sarcoidosis are limited, its pathological features and underlying principles suggest opportunities for early intervention against adverse progression. When monocytes are in a “pre-trained” or “pre-stimulated” state, secondary stimulation may trigger persistent hyperactivation, leading to enhanced inflammation, multinucleated macrophage formation, and granuloma maintenance. This process may further promote tissue fibrosis, resulting in damage and functional impairment. Thus, while trained immunity may enhance host defense, it can also drive chronic inflammation. Further studies are needed to clarify the potential detrimental effects of trained immunity in sarcoidosis and to provide mechanistic insights for therapeutic strategies.

#### 5.1.2 Multiple sclerosis

Multiple sclerosis (MS) is a neurodegenerative disorder caused by inflammatory damage to the myelin sheath in the brain and spinal cord. This disrupts neural signal transmission, leading to neurological symptoms, reduced quality of life, and disability. Common symptoms include fatigue, blurred vision, optic neuritis, limb weakness or sensory disturbances, dizziness, balance problems, cognitive impairment, and bladder dysfunction (53). In MS, B lymphocytes exhibit a proinflammatory profile that drives cortical pathology and contributes to neurological dysfunction (54). CD20, a specific surface marker of B cells, is the target of anti-CD20 monoclonal antibodies (e.g., ocrelizumab, ofatumumab, rituximab, ublituximab). These agents selectively deplete B cells through antibody-dependent cellular cytotoxicity and complement-dependent cytotoxicity without impairing antibody production by plasma cells (55, 56). Although anti-CD20 therapy effectively suppresses disease activity, it may trigger reactivation of latent infections (e.g., tuberculosis, hepatitis) and infusion-related reactions (e.g., fever, headache) (57). However, the role of innate immunity remains poorly understood. Comparing peripheral blood from MS patients and healthy controls showed that granulocytes significantly decreased in MS, while monocyte representation remained unchanged (58). CD64^+^ and PD-L1^+^ granulocytes showed no significant decline, whereas other subsets defined by distinct membrane markers displayed marked differences (58). These findings indicate that research on MS pathogenesis and therapy should also focus on elements of the innate immune response. Macrophages in MS patients tend to exhibit a proinflammatory phenotype. Even in the absence of inflammatory stimuli, these cells display increased expression of glycolytic genes, a metabolic feature resembling trained immunity. Additional alterations include downregulation of electron transport chain genes, TCA cycle disruption, and abnormal fatty acid metabolism. These changes impair oxidative metabolism, which is essential for anti-inflammatory macrophage function, and may contribute to or result from the proinflammatory state, perivascular macrophages with elevated glycolytic capacity show enhanced transmigration in MS animal models and are associated with immune cell infiltration (59). This intrinsic pro-inflammatory metabolic program, independent of external signals, suggests that macrophages may be more prone to activation and may even spontaneously generate low-grade inflammation. Such activity can sustain neuroinflammation even in the absence of clear external triggers. These intrinsic defects align with the pathological features of MS and suggest an important role for macrophages. In treated MS patients, levels of key metabolites were similar to those in healthy controls (59). This indicates that therapy has beneficial effects on the energy defects of innate immune cells and shows that macrophage metabolism is closely linked to disease state, possibly influenced by innate immune memory. These findings highlight the importance of macrophages in MS and their potential as therapeutic targets. Normalization of metabolite levels further demonstrates that reversal of this “trained” state is feasible and supports the strategy of targeting metabolic pathways to eliminate maladaptive training.

Epigenetic modifications in immune and glial cells also contribute to MS pathogenesis and progression. Differentially methylated regions and sites have been identified in immune cells and oligodendrocytes from MS patients compared to healthy controls (60). The promoter region of *PADI2*, encoding peptidyl arginine deiminase 2, an enzyme catalyzing myelin basic protein (MBP) citrullination, is hypomethylated in MS. This hypomethylation suppresses MBP production, compromising myelin stability, similar *PADI2* hypomethylation has been observed in PBMCs from MS patients (61). Moreover, hypomethylation of the *IL-17A* promoter in T cells increases IL-17 expression, promoting central nervous system inflammation (62). In PBMCs, citrullination of histone H3 at H3Cit8 prevents heterochromatin protein 1 binding to H3K9me3, thereby suppressing TNF-α and IL-8 expression (63). Histone deacetylation is more prominent in chronic MS lesions than in early-stage lesions, and the efficiency of deacetylation declines as the disease progresses (64). Although current evidence does not establish a direct link between trained immunity and MS, immune cells in MS exhibit epigenetic reprogramming and metabolic changes. These trained-like features may enhance immune activation or induce tolerance, ultimately influencing clinical manifestations. Future studies should further investigate the role of trained immunity in MS, particularly across different disease stages.

#### 5.1.3 Rheumatoid arthritis

Rheumatoid arthritis (RA) is the most common inflammatory arthritis and represents a systemic inflammatory disease that mainly affects the joints (65). In RA, synovial macrophages and T cells excessively secrete TNF-α, driving synovial hyperplasia and inducing IL-6 and IL-1 release, which forms an “inflammatory amplification loop” and leads to cartilage and bone destruction (66). Anti-TNF antibodies such as adalimumab block TNF-α binding to its receptors, reduce inflammatory mediator release, and alleviate inflammation and tissue damage (67). In patients with poor response to TNF inhibitors, anti-CD20 therapy is widely used, improving synovitis, slowing joint destruction, and delaying disease onset in high-risk individuals (68). This raises the question of whether trained immunity could be applied to intervene early in macrophage and T-cell overactivation to block the amplification loop. Increasing evidence indicates that innate immunity plays a critical role in disease initiation and persistence. Circulating monocytes from RA patients produce elevated levels of IL-1β and IL-6 upon *in vitro* stimulation (69), IL-6 contributes to joint destruction (70), while IL-1β promotes osteoclastogenesis, induces matrix metalloproteinase (MMP) expression in chondrocytes, and initiates synoviocyte proliferation (71). Together, these cytokines drive bone degradation. RA monocytes show activation of PI3K/mTOR and MAPK pathways, inhibition of mTOR reduces synovial osteoclast formation and protects against local bone and cartilage damage (72), highlighting the central role of cytokine-driven signaling in RA pathophysiology.

Epigenetic mechanisms also contribute to RA, involving histone acetylation, methylation, and DNA methylation. Increased expression of H3.3 in PBMCs from RA patients has been associated with histone acetylation markers (73). Hypomethylated and hypermethylated genomic regions have been identified in PBMCs, RA synovial fibroblasts, and RA synovial tissues (74–76). LPS stimulation of THP-1 cells from RA patients results in enhanced histone H3 and H4 acetylation at the *CCL2* promoter (77). mTORC1 promotes the expression of lactate dehydrogenase A (LDHA) and Pyruvate kinase isozyme type M2 (PKM2) by activating HIF-1α and catalyzes the reductive carboxylation of glutamine to generate lipid precursors, thereby aggravating synovial inflammation (78, 79). The hypoxic synovial microenvironment further enhances angiogenesis and glycolysis through HIF-1α, creating a vicious cycle of inflammation and hypoxia (80). Dysregulated lipid metabolism in RA is characterized by elevated low-density lipoprotein Cholesterol (LDL-C), impaired high-density lipoprotein cholesterol (HDL-C) function, and abnormal proprotein convertase subtilisin/kexin Type 9 (PCSK9) accumulation, which directly drive inflammation and immune dysregulation. Serum PCSK9 levels correlate positively with Disease Activity Score in 28 Joints and Rheumatoid Factor and accelerate RA progression through dual mechanisms (81). These findings indicate that innate immune cells in RA also undergo metabolic changes resembling trained immunity, sustaining pro-inflammatory responses. In addition, under the context of studying central trained immunity as a basis for inflammatory comorbidities, experimental periodontitis-induced central trained immunity could be transferred to healthy mice via bone marrow transplantation, leading to more severe arthritis in a collagen antibody, induced arthritis model (82). Central trained immunity acted in a maladaptive manner, thereby exacerbating inflammation and increasing the risk of inflammatory comorbidities.

Future research should further investigate the specific mechanisms of trained immunity in RA, particularly how epigenetic reprogramming and metabolic shifts coordinate to regulate monocytes, macrophages, and possibly non-immune cells. Integrating trained immunity–targeted interventions with immunosuppressive therapies may offer more precise and effective treatment strategies for RA patients.

#### 5.1.4 Systemic lupus erythematosus

Systemic lupus erythematosus (SLE) is a chronic, multisystem inflammatory disease caused by abnormal immune activation that attacks self-tissues, representing a diffuse connective tissue disorder. It is an autoimmune disease targeting antigens derived from apoptotic microparticles (MPs) and neutrophil extracellular traps (NETs), and is typically B cell/antibody–driven (83). Conventional treatment relies on glucocorticoids and immunosuppressants, which have substantial side effects. Recently, belimumab and anifrolumab have shown clinical benefits by neutralizing the B cell activating factor to reduce abnormal B cell survival, and by blocking the type I interferon receptor to inhibit interferon-driven inflammation, improving disease activity, reducing flares, and lowering steroid use (84). Monocytes and macrophages are increasingly recognized as key contributors to disease pathogenesis (85). Macrophages from SLE patients exhibit impaired phagocytosis of apoptotic cells and reduced clearance of immune complexes (86). In lupus nephritis, macrophage infiltration serves as a predictor of disease progression (87). Emerging evidence also suggests a possible link between SLE and trained immunity. Circulating monocytes from SLE patients produce higher levels of proinflammatory cytokines in response to Toll-like receptor (TLR) agonists, indicating a trained phenotype. This response is associated with dysregulation of histone H3 lysine 4 trimethylation (H3K4me3) and increased expression of genes involved in metabolism and inflammation (88). However, excessive production of these cytokines may also promote adaptive immune activation, including autoreactive T and B cell responses and antinuclear antibody formation, potentially perpetuating inflammatory tissue damage. BMDMs from lupus-prone mice display hallmarks of trained immunity, such as enhanced mycobacterial killing and increased cytokine production. These functional changes are mechanistically linked to elevated glycolytic metabolism (89). Intraperitoneal injection of β-glucan into 12-week-old NZB/W F1 mice (SLE-prone) exacerbated extramedullary hematopoiesis in the spleen, promoted myeloid skewing, and worsened lupus nephritis, inducing maladaptive trained immunity (90). In SLE mouse models, hematopoietic stem and progenitor cells (HSPCs) exhibited transcriptional reprogramming and myeloid bias. The autoimmune inflammatory environment in SLE may train HSPCs to produce hyperresponsive myeloid cells, further promoting autoantibody generation and organ damage, and potentially driving maladaptive progression (91). Histone modifications have been extensively studied in both human and murine models of SLE (92). For example, elevated histone H3 acetylation and increased H3K4me2 levels at the *CD70* gene promoter are positively correlated with disease activity (93). Aberrant patterns of histone acetylation and methylation have been observed in monocytes and T lymphocytes from SLE patients (94). Therapeutic interventions targeting metabolic and epigenetic processes have demonstrated benefit in SLE, suggesting that inhibition of trained immunity may contribute to these protective effects.

#### 5.1.5 Others

In addition to the autoimmune diseases mentioned above, several skin inflammation related autoimmune or autoinflammatory disorders have also been associated with trained immunity. Such as psoriasis, an inflammatory skin disease, is characterized by erythematous plaques resulting from keratinocyte hyperproliferation (95), although the epigenetic elements of trained immunity in human psoriasis have not been fully defined, studies using imiquimod (IMQ)-induced murine psoriasis models have demonstrated that EpSCs retain innate immune memory by maintaining chromatin accessibility following inflammation. These accessible chromatin regions allow rapid activation of relevant genes upon secondary insult (6). Hyperimmunoglobulin D syndrome (HIDS), a monogenic autoinflammatory disorder, features erythematous rashes and recurrent sterile inflammation. Aberrant trained immunity in HIDS arises from mevalonate kinase pathway dysfunction, leading to metabolite accumulation that activates the AKT–mTOR–HIF-1α axis. This shifts immune cells from oxidative phosphorylation to glycolysis, giving circulating monocytes a persistently activated trained immunity profile (96). This process may further activate the NLRP3 inflammasome, resulting in excessive release of inflammatory cytokines such as IL-1β and amplifying systemic inflammation (97).

### 5.2 Degenerative diseases

#### 5.2.1 Atherosclerosis

Atherosclerotic cardiovascular disease (ASCVD) is a chronic degenerative vascular disease characterized by low-grade, persistent inflammation of the vascular wall, in which monocytes and macrophages play a central role (98). Factors that exacerbate this chronic inflammatory state, including dyslipidemia, obesity, diabetes, hypertension, aging, and smoking, as well as nontraditional risks such as infections and chronic inflammatory diseases, can promote ASCVD progression (99). Trained immunity, through metabolic and epigenetic reprogramming of innate immune cells, enhances inflammatory responses upon secondary stimulation. Given its amplified proinflammatory characteristics, trained immunity may play a key role in infection-related ASCVD. In murine models, short-term exposure to low-dose LPS induces long-lasting monocyte polarization toward a proinflammatory phenotype, aggravating atherosclerotic lesion development (100). Endogenous ASCVD-associated stimuli, such as oxidized low-density lipoprotein (oxLDL), lipoprotein (a), aldosterone, and S100A4 protein, have also been shown to induce trained immunity in monocytes and macrophages. Trained monocytes exhibit enhanced adhesion to vascular endothelium, increased migratory capacity, and elevated inflammatory responses to TLR agonists (101). In trained macrophages, foam cell formation and upregulation of matrix metalloproteinases accelerate ASCVD progression (102).

HSPCs in the bone marrow contribute to trained immunity. Upon primary stimulation, such as infection, vaccination, or inflammation, HSPCs undergo transcriptional and epigenetic reprogramming, generating monocytes and macrophages with enhanced responsiveness. In mouse models of ASCVD (e.g., LDLR^-/-^), a high-fat diet expands bone marrow progenitors and promotes myelopoiesis, accompanied by long-term epigenetic changes linked to NLRP3 inflammasome activation and increased IL-1β secretion. Resulting monocytes adopt a primed state with heightened TLR responses for at least four weeks. Transplanting bone marrow from these trained mice into normal-diet recipients enlarges atherosclerotic lesions (103, 104). Monocytes and macrophages from trained HSPCs produce more proinflammatory cytokines (TNF-α, IL-6, MCP-1) and show enhanced vascular migration, promoting vascular inflammation and ASCVD progression (105). Hypercholesterolemia also drives HSPC proliferation and myeloid skewing, producing proinflammatory, pro-atherogenic myeloid cells (105).

Clinical observations support these findings. Using 18F-fluorodeoxyglucose positron emission tomography, researchers have detected significantly elevated glucose metabolism in human atherosclerotic plaques, particularly within lipid-rich necrotic cores and regions densely infiltrated by immune cell (106, 107), high-risk plaques, in particular, display increased glycolytic activity (108), and as a metabolic hallmark of trained immunity (109, 110). Although the direct involvement of trained immunity remains to be confirmed, high-risk plaques produce higher levels of proinflammatory cytokines and chemokines than low-risk, stable plaques (108). Furthermore, circulating monocytes isolated from symptomatic ASCVD patients exhibit a more proinflammatory phenotype compared to those from healthy individuals (111).

In addition to immune cells, vascular non-immune cells, such as endothelial cells and smooth muscle cells (SMCs), also participate in trained immunity during atherosclerosis. Vascular SMCs isolated from diabetic mouse models exhibit enhanced migratory capacity, upregulated expression of inflammatory genes, and increased adhesion to monocytes. These trained-like features persist over time and remain detectable even *in vitro* (112). Similarly, stimulation with oxLDL induces sustained proinflammatory “priming effects” in cultured coronary artery SMCs (113). These findings indicate that SMCs, like innate immune cells, can undergo trained immunity and maintain a long-term activated state. Endothelial cells also exhibit similar behavior. The pro-atherogenic lipid molecule lysophosphatidylcholine reprograms aortic endothelial cells, inducing sustained inflammatory activation (114). High glucose levels drive endothelial cells into a prolonged proinflammatory state, characterized by NF-κB upregulation and increased expression of pro-atherogenic genes such as MCP-1 and VCAM-1 (115). These “trained” vascular non-immune cells may play critical roles in the progression of atherosclerosis, particularly by promoting vascular inflammation and accelerating ASCVD development.

Treatment of atherosclerosis focuses on lowering blood lipids, controlling blood pressure, improving glucose metabolism, and using antiplatelet agents to slow disease progression (116). Anti-inflammatory approaches, such as the IL-1β inhibitor canakinumab in the CANTOS trial, have also been used to reduce cardiovascular events (117). Although these strategies lower event rates, they do not address chronic low-grade vascular inflammation and fail to fully halt atherosclerosis progression. Given these limitations, targeting trained immunity provides a novel therapeutic perspective. Interventions aimed at modulating trained immune responses in both immune and non-immune vascular cells may offer more effective approaches for the treatment of atherosclerosis.

#### 5.2.2 Alzheimer’s disease

Alzheimer’s disease (AD) is the most common form of dementia, characterized by progressive cognitive decline and behavioral impairment, with risk increasing with age. It is an inflammatory and neurodegenerative disorder driven by extracellular accumulation of amyloid-β (Aβ) and intracellular aggregation of Tau protein (118). Aβ deposition activates microglia, triggering local inflammation and further promoting plaque formation (119). Current therapies include anti-Aβ monoclonal antibodies, anti-Tau treatments, and TREM2 agonists. Lecanemab, a humanized monoclonal antibody targeting the N-terminus of Aβ, reduces neurotoxicity by promoting Aβ clearance (120). Anti-Tau therapies target Tau’s N-terminus to inhibit abnormal aggregation and phosphorylation, slowing neurodegeneration. TREM2 agonists enhance microglial phagocytosis, promoting Aβ and Tau clearance and improving the neural microenvironment (121). However, microglia, the resident immune cells of the brain, normally play a protective role. In early AD, they secrete neurotoxic cytokines, sustaining chronic inflammation (122). In APP23 transgenic mice, a single intraperitoneal LPS injection (1×LPS) at 3 months increases Aβ plaque burden and total Aβ while reducing brain IL-10. Gene co-expression analysis shows upregulation of HIF-1 signaling and glycolysis-related genes. Microglia display higher mitochondrial membrane potential and lactate release, reflecting HIF-1α activation and a metabolic shift toward glycolysis. In contrast, repeated LPS injections (4×LPS) produce opposite effects, indicating that trained immunity exacerbates AD pathology, whereas immune tolerance mitigates it (123). In another model expressing mutant human APP, presenilin, and Tau, repeated LPS increases Tau phosphorylation without significantly changing Aβ deposition (124). These findings suggest that trained immunity contributes to neuroinflammation and neurodegeneration in AD. In early or preclinical stages, it is marked by heightened inflammatory responses and increased Aβ production, potentially causing neuronal damage. In later stages, chronic Aβ exposure or inflammation may induce tolerance, reducing cytokine release and promoting repair, though this response can be maladaptive or insufficient. Notably, microglia from 1×LPS- and 4×LPS-treated mice exhibit distinct levels of H3K4me1 and H3K27ac, suggesting that epigenetic remodeling plays a key role in trained immunity–associated microglial plasticity (123). Other myeloid cells, including DCs and neutrophils, may also participate in AD-related neuroinflammation and degeneration. These cells may even infiltrate the central nervous system across the blood–brain barrier and contribute to neuronal injury and cognitive decline (125–128). Collectively, AD progression may be driven by complex interactions among multiple immune cell types, with trained immunity and its epigenetic imprinting contributing to disease dynamics across different stages.

Epigenetic regulation plays a critical role in the progression of AD. Demethylation at the promoter region of the *APP* gene has been implicated in Aβ accumulation in the aging brain (129). Methylation changes in the *Tau*, particularly at CpG dinucleotide sites, have been shown to alter microtubule function, promoting aberrant Tau aggregation and the formation of neurofibrillary tangles (130, 131). In addition, hyperphosphorylated Tau can suppress gene expression by recruiting histone deacetylases (HDACs) to condense chromatin structure (129). Among HDAC family members, SIRT1 catalyzes the deacetylation of lysine 28 on Tau, inhibiting its normal function and facilitating aggregation (132). Decreased SIRT1 expression in the cortex is closely associated with the accumulation of both Aβ and Tau in AD patients (133), aberrant nuclear–cytoplasmic localization of H3K4me3 has also been reported in early-stage AD (94), though its functional significance remains unclear (134). These findings suggest that early epigenetic alterations may contribute to AD pathology.

Immune cells in AD may undergo increased aerobic glycolysis, a key metabolic driver of trained immunity. Under LPS and IFN-γ stimulation, microglial metabolism shifts toward glycolysis and the PPP (135). Metabolic dysregulation in microglia impairs Aβ clearance and sustains proinflammatory cytokine release (136). In AD animal models, mTOR signaling is activated early, whereas TREM2-deficient mice show mTOR pathway defects, leading to abnormal ATP levels and biosynthetic pathways (137). Although direct evidence of trained immunity in clinical AD is still limited, transcriptional, epigenetic, and metabolic alterations observed in immune cells, including microglia, monocytes, and dendritic cells, from both patients and animal models are consistent with mechanisms underlying innate immune memory. These insights support the hypothesis that trained immunity–like processes may contribute to the immunopathogenesis of AD.

### 5.3 Pneumonia

Pneumonia is a lung infection caused by bacteria, viruses, or other microorganisms, primarily affecting the alveoli. Among these, bacterial and viral pneumonia are the most common. Recent studies have linked both forms to trained immunity. *S. pneumoniae* is the leading cause of community-acquired pneumonia and remains a life-threatening pathogen (138). Infection with *S. pneumoniae* induces histone H3K4me2 modifications in respiratory epithelial cells, which persist for at least nine days after bacterial clearance by antibiotics, these epigenetic changes result in altered cellular metabolism and lysosomal transport, enhancing bacterial adhesion and promoting secondary infections (28). Similarly, LPS exposure induces long-term phenotypic changes and trained immunity in lung-resident macrophages, this response provides significant protection against *S. pneumoniae* but offers limited defense against SARS-CoV-2 (23). In the context of viral infections, influenza virus has been shown to induce trained immunity in alveolar macrophages, conferring long-lasting anti-tumor immunity. This effect persists for at least 30, 60 and even 120 days after infection, significantly inhibiting pulmonary tumor growth. The protective mechanism depends on IFN-γ, NK cells, and epigenetic and metabolic reprogramming (139). SARS-CoV-2 infection–induced pneumonia may also be associated with trained immunity. SARS-CoV-2, the causative agent of COVID-19, is transmitted primarily through respiratory droplets, similar to influenza. Clinical manifestations range from asymptomatic infection and mild upper respiratory symptoms to severe pneumonia with respiratory failure and death (140). By enhancing innate immune responses via epigenetic and metabolic reprogramming, trained immunity may provide early antiviral protection in COVID-19, reducing viral replication and inflammation. Interestingly, *S.aureus* skin infections can induce eosinophil-mediated innate immune memory via IL-33 and C5a, leading to bone marrow reprogramming. This alters mature myeloid cell function and exacerbates lung inflammation in systemic allergy models (141). Future research should explore the role of trained immunity in pneumonia of different etiologies. Understanding how epigenetic and metabolic reprogramming modulate immune responses may offer new therapeutic strategies for the prevention and treatment of pneumonia.

### 5.4 Gastrointestinal inflammation

Studies on trained immunity in gastrointestinal inflammation primarily focus on intestinal inflammation and pancreatitis. During maternal infection, elevated IL-6 crosses the placenta and acts on fetal intestinal epithelial cells. This proinflammatory signal induces epigenetic changes, promoting Th17 differentiation and expansion, enhancing resistance to intestinal infection but increasing risk of intestinal inflammation. These modifications include lasting changes in chromatin accessibility and transcriptional programs in intestinal epithelial stem cells, which may persist into adulthood (9). The integrity of the intestinal barrier is essential for maintaining host health. When this barrier is disrupted, commensal bacteria can translocate systemically and trigger inflammatory responses. One study showed that barrier damage allows *Enterococcus faecalis*, a member of the gut microbiota, to migrate to the bone marrow, where it induces trained immunity in myeloid progenitors via the Mincle receptor. This response may be either protective or detrimental, depending on context. Mice treated with heat-killed E. faecalis exhibited less weight loss and higher survival rates following infection with *Candida albicans* or influenza virus. However, in inflammatory models, trained immunity may aggravate disease, for instance, in dextran sulfate sodium-induced colitis, Mincle-deficient mice displayed milder pathological symptoms, indicating that trained immunity contributes to the pathology of inflammatory bowel disease (142). These findings offer novel insights into the complex interplay among the gut microbiota, immune system, and inflammation, which may inform new immunotherapies and improve understanding of diseases related to increased gut permeability. Moreover, inflammation is a major risk factor for pancreatic ductal adenocarcinoma. During pancreatitis, oncogenic kirsten rat sarcoma viral oncogene homolog (KRAS) mutations accelerate tumor progression. Notably, even transient inflammatory episodes can sensitize pancreatic epithelial cells to subsequent KRAS-driven transformation. This adaptive response involves persistent transcriptional and epigenetic reprogramming, enabling rapid activation of ADM during recurrent inflammation, thereby limiting tissue damage (31).

### 5.5 Allergic diseases

Allergic diseases have emerged as a global public health challenge, with rising prevalence imposing a significant socioeconomic burden. Chronic conditions such as allergic asthma, allergic rhinitis, food allergy, atopic dermatitis, and anaphylaxis impair quality of life and contribute to substantial healthcare expenditures (143). The treatment of allergic diseases has shifted from symptomatic control to a strategy combining immune modulation and targeted therapy. Allergen immunotherapy and biologics/monoclonal antibodies are emerging as key options, offering not only symptom relief but also the potential to induce tolerance and modify disease progression (144). Traditionally, allergic responses were thought to be driven primarily by the adaptive immune system, particularly IgE-mediated reactions. However, recent studies suggest that aberrant activation of the innate immune system also plays a critical role in the onset and progression of allergic diseases. Notably, trained immunity has been proposed as a potential mechanism. Early-life innate immune hyperactivation has been observed in allergic children, while asthmatic children display immune dysregulation characterized by reduced IFN-γ production and ILC2 expansion in response to rhinovirus or LPS stimulation (145). These phenomena may result from allergen- or virus-induced trained immunity, leading to long-lasting immune dysfunction and increased susceptibility to allergic inflammation. In allergic mouse models or patients with house dust mite–induced asthma, macrophages exhibit exaggerated production of TNF-α, CCL17, leukotrienes, PGE2, and IL-6 upon stimulation. This process depends on the TNF signaling pathway and involves 2-hydroxyglutarate accumulation and KDM1A-mediated demethylation, identifying potential therapeutic targets for allergic asthma (146). In a study using single-cell RNA sequencing, Li et al. (2022) identified an inflammatory neutrophil subpopulation in allergic asthma with molecular features of innate immune memory. Asthma may reprogram neutrophil populations, leading to expansion of G-CSFR^+^FcγRIIb^+^ neutrophils, which may represent memory-like neutrophils and serve as novel targets in neutrophil-dominant asthma (147). Two experimental studies further indicate that infection-induced reprogramming of innate immune cells may prevent asthma development. In one model, murine gammaherpesvirus 4 lung infection suppressed HDM-induced experimental asthma (HDM-EAA) through two mechanisms (1): downregulating costimulatory molecules CD80/CD86 on migratory dendritic cells, impairing Th2 activation; and (2) inducing embryonically derived alveolar macrophage apoptosis, while recruiting bone marrow–derived monocytes that differentiated into anti-inflammatory macrophages with sustained IL-10 production. These monocytes persisted in lung parenchyma for up to 28 days, suggesting the formation of long-term trained immunity via epigenetic reprogramming (148). In another study, neonatal peritoneal inoculation of EV-A71 virus induced glycolysis-dependent proinflammatory training in macrophages, these trained cells displayed prolonged (≥21 days) secretion of IL-6, TNF-α, and CCL17 and aggravated HDM-EAA. Adoptive transfer of EV-A71–trained BMDMs into naïve mice enhanced Th2 inflammation and allergen-specific IgE levels. Further analysis revealed temporal polarization of these trained macrophages: an early Th2-skewed phase, followed by a classical proinflammatory phenotype. This process was abrogated by the glycolysis inhibitor 2-deoxy-D-glucose (2-DG), confirming the central role of metabolic reprogramming in immune training (148). In addition to immune cells, other cell types such as airway epithelial cells may also contribute to trained immunity in allergic disease. Airway epithelial cells play key roles in asthma pathogenesis and respond to microbial compounds, allergens, and pollutants. Single-cell transcriptomic profiling in chronic rhinosinusitis revealed basal epithelial cells with Th2 cytokine memory characteristics, suggesting that epithelial cells may act as allergen memory reservoirs. Notably, microbial imprinting in the respiratory epithelium may dynamically regulate future immune responses by modulating cytokine output such as IL-1b and IL-8 (149), resembling the trained immunity seen in macrophages. Beyond asthma, food allergy has gained attention due to rising prevalence in Western countries. Trained immunity may influence food allergy development by reshaping the DCs–T cell interaction network and promoting Th2-skewed responses. In peanut-allergic infants, PBMCs exhibit excessive TNF-α production upon nonspecific stimulation. This hyperresponsive immune phenotype persists in allergic adolescents, marked by increased circulating dendritic/monocyte populations and elevated secretion of IL-6, IL-1β, and TNF-α upon LPS challenge (150). In summary, trained immunity provides a novel conceptual framework for understanding allergic diseases. It offers promising avenues for early intervention and the development of targeted therapies based on epigenetic and metabolic reprogramming of immune and non-immune cells.

Trained immunity exerts long-lasting effects on cell function through epigenetic regulation and metabolic reprogramming (**Table 1**) and shows bidirectional roles in immune regulation: disease-induced trained immunity can, in turn, exacerbate pathology, creating a self-reinforcing vicious cycle. Accordingly, new prevention and treatment strategies should aim to enhance its beneficial effects and intervene early in harmful reprogramming. These findings offer new perspectives for targeted therapies, though underlying mechanisms and clinical applications require further investigation.

**Table 1:** Trained immunity in inflammatory diseases: from metabolic dysregulation to epigenetic reprogramming.

Disease	Involved cell types	Metabolic changes	Epigenetic reprogramming	Functional consequences	References	Potential therapeutic strategies
Atherosclerosis	Monocytes; Macrophages; vascular endothelial cells; smooth muscle cells; HSPCs	Increased glycolysis (high-risk plaques), impaired oxidative phosphorylation	NLRP3 inflammasome-associated epigenetic reprogramming	Enhanced pro-inflammatory response, increased endothelial adhesion and migratory capacity, foam cell formation and expression of matrix metalloproteinases, exacerbated vascular inflammation	(101–104, 108, 113, 114)	Targeting metabolism (e.g., glycolysis inhibitors) or epigenetic modifications (e.g., HDAC inhibitors)
Sarcoidosis	Monocytes; Macrophages	mTOR pathway activation, dysregulation of glycolysis and the tricarboxylic acid cycle	abnormal methylation of genes such as *HLA-DPB2 and CXCL7*	Granuloma formation and maintenance, increased secretion of pro-inflammatory cytokines (TNF-α, IL-6)	(44–47, 51, 52, 219, 220)	mTOR inhibitors (e.g., rapamycin), JAK/STAT pathway inhibitors
Alzheimer’s disease	Microglia; dendritic cells; neutrophils	HIF-1α signaling activation, enhanced glycolysis	abnormal methylation of the *Tau*, downregulation of *SIRT1*, mislocalization of H3K4me3	Increased Aβ deposition, exacerbated neuroinflammation, cognitive decline	(94, 119, 125–128, 133, 149)	Targeting glycolysis or epigenetics (e.g., SIRT1 activators)
Multiple sclerosis	Macrophages; Oligodendrocytes	Increased glycolysis, reduced oxidative metabolism	Hypomethylation of *PADI2*, demethylation of the *IL-17A* promoter, histone deacetylation	Myelin destruction, central nervous system inflammation	(59–62, 221–223)	Metabolic regulation (e.g., restoration of oxidative phosphorylation), epigenetic interventions (e.g., methylation inhibitors)
Rheumatoid arthritis	Macrophages; synovial cells	PI3K/mTOR activation; upregulation of glycolytic rate-limiting enzymes (PKM2, HK2)	Histone H3/H4 acetylation (e.g., *CCL2* promoter), abnormal DNA methylation	Joint destruction, increased release of pro-inflammatory cytokines (IL-1β, IL-6)	(70–72, 77, 224–227)	mTOR inhibitors, targeting metabolic reprogramming (e.g., 2-DG)
Systemic lupus erythematosus	Monocytes; macrophages	Increased glycolysis, suppressed oxidative phosphorylation	High histone H3 acetylation and elevated H3K4me2 levels at the *CD70* gene promoter are positively correlated	Autoantibody production, tissue inflammation (e.g., lupus nephritis)	(86, 90, 93)	Glycolysis inhibitors, HDAC inhibitors
Pneumonia	Alveolar macrophages; respiratory epithelial cells	Metabolic reprogramming (altered glycolysis/oxidative phosphorylation balance)	H3K4me2 modification (persisting after *Streptococcus pneumoniae* infection)	Increased susceptibility to secondary bacterial infection or enhanced antiviral protection	(23, 28, 140)	Epigenetic interventions (e.g., regulation of histone methylation)
Gastrointestinal inflammation	Intestinal epithelial cells; myeloid progenitor cells	Glycolysis-dependent pro-inflammatory phenotype	Altered chromatin accessibility (e.g., in offspring intestinal stem cells following maternal infection)	Disruption of the intestinal barrier, increased risk of inflammatory bowel disease	(9, 31, 142)	Targeting the Mincle receptor or modulation of the microbiota
Allergic diseases	Macrophages; neutrophils; airway epithelial cells	Glycolysis-dependent inflammation (e.g., accumulation of 2-hydroxyglutarate)	KDM1A-mediated histone methylation	Enhanced Th2 immune response, excessive production of allergic mediators (e.g., IgE, leukotrienes)	(146–148)	Metabolic interventions (e.g., 2-DG), targeting epigenetic enzymes (e.g., KDM1A inhibitors)

HSPCs, Hematopoietic Stem and Progenitor Cells; 2-DG, 2-Deoxy-D-glucose; HDAC, Histone Deacetylase; SIRT1, Sirtuin 1; PAD2, Peptidyl Arginine Deiminase 2; KDM1A, Lysine Specific Demethylase 1; Aβ, amyloid-beta.

## 6 Treatment of inflammatory diseases based on training immune mechanisms

**Figure 3:**
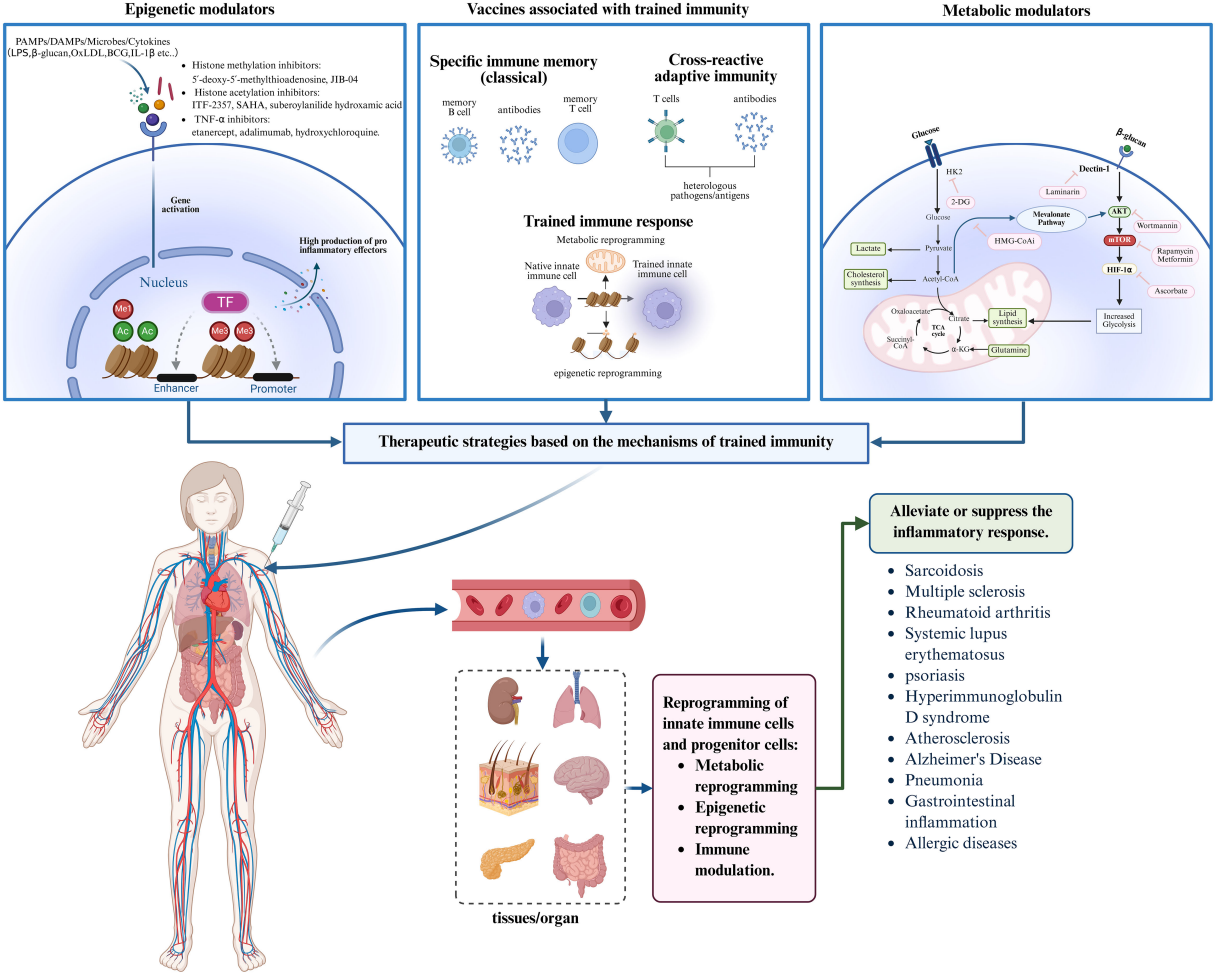
Trained immunity-based therapeutic strategies for inflammatory diseases. Trained immunity offers novel intervention strategies for inflammatory diseases. By developing epigenetic modulators, metabolic regulators, and trained immunity-related vaccines, these agents can be administered systemically and delivered via the bloodstream to target organs. This approach aims to modulate trained immunity through epigenetic remodeling, metabolic reprogramming, and immune activation, thereby effectively alleviating or suppressing inflammatory responses and improving disease outcomes. Created with BioRender.com.

For inflammatory diseases, most current therapies target the adaptive immune system or provide symptomatic relief by suppressing inflammatory mediators. However, these approaches often fail to prevent disease recurrence or chronic progression. The discovery of trained immunity opens new therapeutic avenues by enabling interventions that target excessive innate immune activation. By modulating epigenetic remodeling and metabolic reprogramming within trained immunity pathways, it is possible to develop targeted therapies that reprogram inflammatory memory in myeloid cells at its source. In addition, regulatory vaccines and smart nanomaterials designed to modulate trained immunity, particularly those targeting bone marrow or inflamed tissues, offer novel, precision-based strategies for inflammatory disease management. These approaches hold promise for achieving long-term immune tolerance and sustained inflammation resolution (**Figure 3** **).**

### 6.1 Based on epigenetic reprogramming

Unlike classical adaptive immune memory, which relies on antigen receptor gene rearrangement, trained immunity operates through epigenetic modifications that increase chromatin accessibility at gene loci following primary stimulation. Upon secondary exposure, this enables rapid transcriptional activation of genes involved in inflammation, antimicrobial defense, and stress responses, without altering the underlying DNA sequence. In eukaryotic cells, genomic DNA is compacted into nucleosomes by structural histone proteins, rendering most gene regions inaccessible to transcription factors. Transcription initiation requires nucleosome displacement at promoter regions to expose regulatory elements, allowing transcription factor binding and recruitment of RNA polymerase (74, 151). Chromatin accessibility is regulated by epigenetic modifications, including histone methylation, histone acetylation, and DNA methylation, among which histone modifications are well-established drivers of inflammatory memory.

For instance, IFN-γ stimulation of HeLa cells increases H3K4me2 at the DRA promoter, which persists after withdrawal and across multiple cell cycles (152). Similarly, Primary *S. pneumoniae* infection induces H3K4me2 enrichment in respiratory epithelial cells (28). In murine EpSCs, inflammatory signals activate STAT3, which translocates to the nucleus and binds H3K4me1- and H3K27ac-marked chromatin, establishing an epigenetically poised state (6). Histone modifications also mediate trained immunity *in vitro*: in β-glucan–trained monocytes, promoters and enhancers of inflammation- and immunity-related genes gain H3K4me1, H3K4me3, and H3K27ac marks, maintaining transcriptionally active chromatin and enabling enhanced cytokine production upon restimulation (153). In contrast, LPS-induced tolerance is marked by attenuated transcriptional responses upon secondary LPS challenge. Tolerized macrophages fail to deposit active histone marks (e.g., H3K4me1, H3K27ac) at the promoters of tolerant genes, resembling the immune paralysis seen in sepsis, interestingly, β-glucan injection can reverse LPS-induced tolerance by restoring H3K27ac deposition and reactivating macrophage function. The BET family small-molecule inhibitor I-BET151 (GSK1210151A) is primarily used to suppress inflammation and for anti-tumor purposes. Evidence supporting its role as an mTOR inhibitor remains largely preclinical (154). Its limitation is the inability to reverse effects once tolerance is established, making it potentially ineffective in monocytes that have already experienced inflammatory activation. Nonetheless, β-Glucan and I-BET151 offer a theoretical basis and potential targets for developing immunomodulatory strategies against tolerance-related diseases such as sepsis (155).Similarly, BCG-induced trained immunity involves the remodeling of H3K9me3, H3K4me3, and H3K27ac histone marks (156, 157). Genome-wide H3K27ac ChIP-seq analyses revealed enrichment of this active histone modification at genes encoding the oxLDL receptor, a marker of atherosclerosis, and other inflammation-related genes in monocytes following BCG vaccination, suggesting transcriptional activation of these pro-atherogenic genes (157). The trained phenotype and gene-specific H3K4me3 enrichment persist for at least three months and up to one year post-vaccination (156). In atherosclerosis, oxLDL-induced trained immunity in monocyte-derived macrophages also promotes H3K4me3-mediated activation of genes involved in inflammation, chemotaxis, and foam cell formation (102). The histone methyltransferase inhibitor 5′-deoxy-5′-methylthioadenosine (MTA) reverses histone methylation and prevents chromatin remodeling, effectively abrogating the trained immunity phenotype induced by oxLDL. This finding offers a potential strategy for modulating inflammation in atherosclerosis (102, 158, 159). Histone methylation is regulated by a complex network of enzymes, including histone methyltransferases and demethylases, which modulate gene expression by adding or removing methyl groups at specific histone residues (160). The broad-spectrum Jumonji histone demethylase inhibitor JIB-04 reduces trained immunity by modulating the repressive H3K9 mark (161). To date, evidence for its therapeutic effects as an mTOR inhibitor is limited to pharmacological and animal studies, with no established clinical trials (162). Similarly, histone acetylation, catalyzed by histone acetyltransferases (HATs) and HDACs, plays a regulatory role in trained immunity (163). Class I/II HDAC inhibitors used in cancer therapy, including vorinostat (suberoylanilide hydroxamic acid) and others such as ITF-2357 and SAHA, have shown anti-inflammatory effects in inflammatory diseases (164). Vorinostat is not a classical mTOR antagonist but has been used clinically in combination with mTOR inhibitors or to modulate mTOR-related pathways (165). ITF-2357 has advanced in multiple clinical trials, with recent progress in some indications; for example, a phase II study (NCT00928707) showed that SAHA combined with hydroxyurea was well tolerated (166). In trained monocytes, elevated NAD^+^/NADH ratios and lactate accumulation modulate HDAC activity and influence downstream gene expression (167, 168). In THP1 cells from rheumatoid arthritis patients, LPS stimulation increases H3 and H4 acetylation at the *CCL2* promoter, which can be reversed by TNF-α inhibitors such as etanercept and adalimumab (77). Besides. In SLE, hydroxychloroquine suppresses trained immunity by inhibiting H3K27ac and H3K4me3 modifications at inflammation-related genes (169).

Although less studied, DNA methylation also contributes to trained immunity. In human embryonic kidney cells, persistent TNF-α stimulation activates TET enzymes, which demethylate the *CALCB* enhancer and *IL32* promoter, establishing a trained-like transcriptional state (170). In trained macrophages, long-term changes in gene expression are associated with altered DNA methylation patterns, particularly 5-methylcytosine (171). Whether modulating DNA demethylation can similarly correct inflammatory dysregulation remains an open question. Certain metabolites, including fumarate, α-ketoglutarate (α-KG), and succinate, regulate TET family demethylase activity, although direct DNA methylation changes have not been consistently observed in trained immunity models (50, 172). In conclusion, both histone modifications and DNA methylation serve as central regulatory mechanisms in trained immunity. Targeting these epigenetic pathways may offer a precise strategy to control excessive trained immune responses and alleviate inflammation-related diseases.

### 6.2 Based on metabolic reprogramming

Trained cells not only enhance gene expression via epigenetic remodeling but also reprogram metabolic pathways to meet elevated energy demands. In quiescent states, immune cells primarily rely on oxidative phosphorylation (OXPHOS) and fatty acid oxidation (FAO) for energy. Upon activation, monocytes and macrophages shift toward glycolysis, lipid metabolism, and amino acid metabolism to support proinflammatory functions.

Glycolysis is intrinsically upregulated in the trained immunity process, independent of external stimuli (173). β-glucan-trained monocytes exhibit increased glucose uptake and lactate production, reflecting enhanced glycolytic flux (110). Similarly, BCG-trained monocytes show marked increases in glycolysis and glutaminolysis. Pharmacological or genetic inhibition of key glycolytic enzymes impairs trained immunity (109). The Akt/mTOR/HIF1α axis orchestrates this metabolic reprogramming by modulating HIF1α activity and glycolytic enzyme expression (174, 175). Similarly, inhibition of Akt/mTOR/HIF1α signaling suppresses β-glucan and BCG-induced trained immunity (109, 110). 2-DG, and metformin are established mTOR inhibitors. Studies using rapamycin-loaded high-density lipoprotein nanoparticles (mTORi-HDL) showed that they prevent post-transplant monocytes from developing trained macrophages upon exposure to alloantigens and HMGB1. mTORi-HDL accumulates in the graft and is taken up by myeloid cells; three intravenous injections reduce inflammatory cell infiltration, prolong graft survival, and suppress macrophage proinflammatory responses (176). A phase I trial determined that 2-DG combined with docetaxel at 63 mg/kg/day is clinically tolerable, showing manageable toxicity and modest antitumor activity (177). Experimental studies indicate that metformin activates Adenosine 5’-monophosphate (AMP)-activated protein kinase and/or inhibits mTORC1 via pathways including regulated in development and DNA damage responses 1 (178). Besides, mTOR activation occurs on lysosomal surfaces (179), where trained immunity is marked by lysosomal gene activation (155). Lysosomotropic agents like chloroquine and hydroxychloroquine impair lysosomal function and robustly inhibit trained immunity (169). The mTOR metabolic signaling pathway regulates HIF-1α activity. In HIF-1α knockout mice, the absence of HIF-1α signaling results in defective epigenetic programming and impaired production of proinflammatory cytokines (180). Succinate, a glycolytic intermediate, stabilizes HIF-1α by inhibiting its degradation, thereby sustaining chromatin accessibility. As an epigenetic modulator, succinate also suppresses histone and DNA methylation, supporting prolonged expression of glycolysis-related genes (109, 181). Collectively, the Akt/mTOR/HIF-1α axis coordinates metabolic and epigenetic reprogramming to drive and maintain trained immunity, representing a potential target for therapeutic intervention. In β-glucan–trained cells, the PPP is also upregulated. However, PPP inhibition has minimal impact on trained immunity, suggesting a limited role in this process (50). Several metabolism-targeting drugs are used not only in immune-related conditions such as cancer but also in inflammation research. 2-DG and dichloroacetic acid inhibit glycolysis and effectively block trained immunity induction *in vitro* (182). Glyceraldehyde 3-phosphate dehydrogenase (GAPDH), a key glycolytic enzyme, also exerts immunomodulatory effects. Through metabolic reprogramming, GAPDH regulates trained macrophages and reduces allergen-induced airway inflammation in murine models of allergic asthma. Mechanistically, GAPDH promotes the transition of M2 macrophages to a proinflammatory M1 phenotype, thereby suppressing Th2 cell activation and exerting anti-inflammatory effects (183).

In addition to glycolysis, enhanced glutamine metabolism is a critical metabolic feature of trained immunity. Inhibition of glutaminase significantly suppresses β-glucan and BCG-induced trained immunity (50, 109). Both stimuli promote glutaminolysis through HIF-1α activation, increasing intracellular α-KG levels. α-KG enters the TCA cycle, supplying energy to support cell function (184). Supplementation with glutamine, arginine, and tryptophan further enhances immune responses in trained cells. These amino acids contribute to DNA synthesis, redox homeostasis, protein translation, and nitric oxide production, thereby improving innate immune cell function (185, 186).

Lipid metabolism also plays a vital role in trained immunity. β-glucan-induced training depends on mevalonate, a key intermediate in the cholesterol biosynthesis pathway. Monocytes from patients with HIDS, characterized by mevalonate accumulation, exhibit a trained immunity, like phenotype. Statins, which inhibit mevalonate production, effectively block β-glucan-induced training (50). Additionally, statins and liver X receptor antagonists suppress cholesterol biosynthesis and reduce mevalonate availability, thereby preventing mTOR-mediated trained immunity (96, 187). In addition, fluvastatin, an inhibitor of the rate-limiting enzyme HMG-CoA reductase, also attenuates β-glucan–induced training *in vitro* (184). In contrast, aldosterone promotes trained immunity by enhancing fatty acid synthesis, an effect that can be blocked by aldosterone receptor antagonists (188). Lipid signaling also regulates PPARs, SREBPs, mTOR, and various lipid mediators, modulating the metabolic state, inflammatory response, and functional output of trained immune cells (189–192). In summary, metabolic reprogramming in trained immunity involves the coordinated regulation of glucose, amino acid, and lipid metabolism. These pathways not only meet the energetic demands of immune activation and inflammation but also shape long-term gene expression through epigenetic mechanisms. Regulation of key signaling pathways such as HIF-1α, mTOR, and Akt, along with control over amino acid and lipid metabolism, provides a promising framework for developing therapeutic strategies against trained immunity–associated hyperinflammatory diseases.

### 6.3 Vaccines about trained immunity

Conventional vaccines primarily rely on the stimulation of antigen-specific T and B cells to elicit adaptive immune responses against specific pathogens. With growing insights into trained immunity, several classical vaccines have been shown to induce long-lasting functional reprogramming of innate immune cells, resulting in enhanced nonspecific protection upon subsequent infections. MV130, a sublingually administered inactivated polybacterial vaccine composed of 90% Gram-positive and 10% Gram-negative bacteria (193), rapidly induces DCs to secrete proinflammatory cytokines such as IL-6, TNF-α, and IL-1β. It also promotes Th1 and Th17 responses against both intracellular and extracellular pathogens, followed by IL-10 production to limit excessive inflammation (194). In a virus-induced asthma model, MV130 demonstrated nonspecific protection by enhancing neutrophil-mediated immunomodulation, thereby preventing asthma exacerbation during rhinovirus infection (195). A phase III randomized placebo-controlled trial involving 120 wheeze-prone children showed that daily sublingual administration of MV130 for six months significantly reduced wheezing episodes, symptoms, and medication use, with protective effects lasting another six months (196). In addition, allergen–mannan conjugate vaccines have been shown to reprogram monocytes into tolerogenic DCs via epigenetic and metabolic remodeling, supporting the induction of trained tolerance in allergen immunotherapy (197).

Vaccines typically consist of antigens and adjuvants. Due to the weak immunogenicity of antigens alone, adjuvants are essential to amplify and sustain immune responses (198). Many live-attenuated or inactivated vaccines act as self-adjuvants by stimulating PRRs. BCG is a classic example. BCG induces trained immunity via chromatin remodeling and metabolic rewiring, enhancing the functionality of monocytes and neutrophils. Upon secondary exposure to unrelated pathogens, trained cells exhibit increased cytokine production and antimicrobial capacity (199–201). A phase III clinical trial (NCT03296423) demonstrated that BCG vaccination enhances inflammatory cytokine secretion via epigenetic modulation, improving resistance to respiratory viral infections in the elderly. BCG also boosts influenza-specific antibody responses and modulates cytokine production in adults (202). Vaccination with AS03-adjuvanted influenza vaccine increases chromatin accessibility in innate immune cells and enhances interferon signaling, conferring broad antiviral resistance *in vitro* against dengue and Zika viruses. These findings suggest that AS03 functions not only as an adaptive immunity enhancer but also as a trained immunity inducer, offering broad-spectrum antiviral potential (203).

At the beginning of the COVID-19 pandemic in 2019, the absence of specific immunity and vaccines drew attention to BCG vaccination. It was considered a potential source of heterologous protection through cross-reactivity with viral antigens, nonspecific activation of lymphocytes, and enhancement of innate immunity via trained immunity. Animal studies have suggested that BCG reduces viral load and mitigated immunopathology in SARS-CoV-2 infection models (204). Unreviewed observational studies also reported lower COVID-19 mortality in countries with universal BCG vaccination, such as South Korea and Japan, compared with countries without such practice, including Italy and the United States (205). However, these associations are indirect and may be confounded by genetic, environmental, vaccine strain, and policy factors. Large-scale studies have generally shown that BCG does not prevent COVID-19 infection or severe disease, and some even indicated an increased risk of symptomatic COVID-19 among vaccine recipients (206–208). The role of BCG in COVID-19 protection remains uncertain, with several unresolved issues. For example, studies in children showed stronger effects when vaccination occurred before 9 months of age, but the influence of age at vaccination on COVID-19 protection is unknown (209). Trained monocytes gradually lose their enhanced cytokine responses, and the duration of heterologous immunity remains unclear (210). It is also unknown whether patients receiving intravesical BCG for bladder cancer gain protection against COVID-19 (211). Overall, the use of BCG in COVID-19 highlights the potential of trained immunity in broad antiviral defense, but faces challenges of inconsistent clinical evidence, uncertain durability, and limited vaccine resources. Other vaccines have also exhibited potential cross-protective effects. Influenza vaccination, by inducing trained immunity, was associated with reduced SARS-CoV-2 infection risk, particularly among healthcare workers and elderly populations (212). Additionally, varicella-zoster virus vaccines may also offer partial protection against COVID-19 (213).

Although trained immunity-based vaccines have not received the same level of global attention as other pandemic countermeasures, multiple clinical trials are ongoing to assess their potential benefits in the context of COVID-19. If proven effective, these vaccines could serve as a proof-of-concept for their use during future pandemics, especially as bridging strategies when pathogen-specific vaccines are unavailable or under development. Given their ability to induce trained immunity, vaccines and vaccine adjuvants should not be viewed solely as passive immunogenic agents. Instead, they represent potential tools for targeted activation of innate immune memory. Through rational antigen design and adjuvant optimization, it may be possible to strategically induce trained immune states to support infection control, immune modulation, and even chronic disease intervention.

### 6.4 Others

Beyond epigenetic and metabolic regulation, advances in nanomedicine have opened new avenues for modulating trained immunity. Nanomaterials not only enable efficient delivery of immunoregulatory agents but can also be engineered to target the bone marrow and myeloid precursors, thereby inducing or suppressing trained immune responses. For example, mTORi-HDL suppress trained immunity in myeloid cells and promote the expansion of anti-inflammatory Ly6C^-^ macrophages, alleviating graft rejection and prolonging graft survival (214). Combined delivery of CD40 co-stimulation inhibitors using TRAF6i-HDL nanoparticles further disrupts both trained immunity and co-stimulatory pathways in myeloid cells, significantly improving transplant acceptance and inducing long-term immune tolerance (176). In addition, bone marrow–targeted nanomaterials engineered for spleen or inflammation site accumulation (e.g., tumors or atherosclerotic plaques) have demonstrated therapeutic potential. These particles either induce monocyte training in the spleen or reprogram myeloid cells at disease sites toward anti-inflammatory phenotypes (215). Although hepatic accumulation of certain nanomaterials may cause adverse effects, targeted delivery to the spleen and inflammatory foci offers promising immunomodulatory and anti-inflammatory benefits (216). In summary, chemical functionalization of nanomaterials allows the modulation of epigenetic and metabolic programs in myeloid progenitors, enabling the induction or inhibition of trained immunity. This strategy holds therapeutic potential for enhancing antimicrobial defense or suppressing maladaptive immune responses in diseases such as atherosclerosis and rheumatoid arthritis. Apart from nanotechnology, molecular targeting therapies are also being explored. IL-1β and GM-CSF are key mediators of trained immunity (180). Monoclonal antibodies against IL-1β have shown efficacy in inflammation-related cardiovascular diseases, reducing thrombotic events and attenuating trained immune responses following myocardial infarction (117). Anti–GM-CSF antibodies are currently under clinical investigation for the treatment of rheumatoid arthritis and related conditions (217). Moreover, RNA interference offers a novel approach to block trained immunity, associated signaling pathways. However, its clinical translation remains in early stages (218).

## 7 Conclusion

Trained immunity, as a novel mechanism of immune regulation based on epigenetic memory in innate immune cells, offers a new framework for understanding the pathophysiology of infectious responses and immune-mediated diseases. Although numerous preclinical and animal studies have provided evidence linking trained immunity to inflammatory diseases, several critical issues remain unresolved. First, more animal models and clinical data are needed to clarify the actual occurrence and mechanistic roles of trained immunity in various inflammatory disorders. Second, strategies to modulate trained immunity must be developed to prevent pathological inflammation caused by excessive stimulation, requiring intervention at the earliest stages of external exposure. Third, the reversible epigenetic modifications and short-lived memory effects of trained immunity offer promising intervention points; however, how to balance therapeutic utilization with the potential risks remains an important research focus. Lastly, identifying ways to integrate innate immune memory with adaptive immune memory may enable the development of more precise and effective therapeutic strategies by harnessing their complementary strengths. To date, the role of trained immunity has been increasingly recognized in SLE, allergic diseases, ASCVD, and MS. However, further studies are needed to elucidate the underlying mechanisms by which trained immunity contributes to disease activity and to determine whether inhibition of trained immunity can prevent disease exacerbation. In contrast, understanding of trained immunity in RA, AD, and sarcoidosis remains limited, although dysregulated metabolic and epigenetic pathways suggest a potential role. Other inflammatory diseases also warrant deeper investigation in this context.

In the future, integrating conventional treatment approaches with emerging immunomodulatory strategies may open new therapeutic avenues for managing complex inflammatory diseases.
